# Dogs Don’t Die Just in Hot Cars—Exertional Heat-Related Illness (Heatstroke) Is a Greater Threat to UK Dogs

**DOI:** 10.3390/ani10081324

**Published:** 2020-07-31

**Authors:** Emily J. Hall, Anne J. Carter, Dan G. O’Neill

**Affiliations:** 1 School of Animal, Rural and Environmental Sciences, Nottingham Trent University, Brackenhurst, Southwell, Notts NG25 0QF, UK; anne.carter@ntu.ac.uk; 2 Pathobiology and Population Sciences, The Royal Veterinary College, Hawkshead Lane, North Mymms, Hatfield, Herts AL9 7TA, UK; doneill@rvc.ac.uk

**Keywords:** VetCompass, primary-care, canine heatstroke, heat-related illness, heat stress, brachycephalic, exertional hyperthermia

## Abstract

**Simple Summary:**

Heat-related illness (often called heatstroke) is a potentially fatal condition inflicted on dogs that will become more common as global temperatures rise. Understanding why dogs develop heatstroke can help to refine prevention strategies through owner education and societal changes. This study aimed to determine the most common triggers of heat-related illness in UK dogs, and which types of dogs were at most risk. We reviewed the veterinary records of over 900,000 dogs and identified that exercise was the most common trigger of heat-related illness in dogs. We also found that heatstroke caused by exercise was just as likely to kill as heatstroke from a hot car. Male dogs and younger dogs were more likely to develop heat-related illnesses triggered by exercise. Older dogs and flat-faced dogs were at greater risk of developing heat-related illness just by sitting outside in hot weather. Any dog can develop heatstroke if left in a hot car, but flat-faced breeds were particularly at risk. As the world gets hotter, we need to include our canine companions in our strategies to stay cool, as they can suffer fatal consequences when we fail to keep them safe.

**Abstract:**

Heat-related illness will affect increasing numbers of dogs as global temperatures rise unless effective mitigation strategies are implemented. This study aimed to identify the key triggers of heat-related illness in dogs and investigate canine risk factors for the most common triggers in UK dogs. Using the VetCompass^TM^ programme, de-identified electronic patient records of 905,543 dogs under primary veterinary care in 2016 were reviewed to identify 1259 heat-related illness events from 1222 dogs. Exertional heat-related illness was the predominant trigger (74.2% of events), followed by environmental (12.9%) and vehicular confinement (5.2%). Canine and human risk factors appear similar; young male dogs had greater odds of exertional heat-related illness, older dogs and dogs with respiratory compromise had the greatest odds of environmental heat-related illness. Brachycephalic dogs had greater odds of all three types of heat-related illness compared with mesocephalic dogs. The odds of death following vehicular heat-related illness (OR 1.47, *p* = 0.492) was similar to that of exertional heat-related illness. In the UK, exertional heat-related illness affects more dogs, and kills more dogs, than confinement in a hot vehicle. Campaigns to raise public awareness about heat-related illness in dogs need to highlight that dogs don’t die just in hot cars.

## 1. Introduction

Heat-related illness (HRI) is a potentially fatal disorder affecting man and animals, and is predicted to become more frequent as climate change increases both the severity and regularity of heatwave events [1]. As mean annual temperatures continue to rise, human populations need to consider mitigation strategies to survive, potentially including migration away from the hottest regions [2] or adaptations to heat including air cooling mechanisms and changes to working practices to reduce the risk of HRI to outdoor workers [3]. Domestic dogs intertwine with every aspect of human society, from providing simple companionship to fulfilling essential working services such as hearing and visual assistant dogs, medical detection dogs and military working dogs [4]. A deeper understanding of the risk factors for canine HRI is therefore urgently needed to ensure adaptations to rising global temperatures can include consideration of canine companions and colleagues.

From a pathophysiological perspective, HRI can be defined as hyperthermia causing progressive systemic inflammation and multi-organ dysfunction, resulting in neurological derangements and potentially death [5]. However, this gives little information on the underlying causes of the clinical event and, more importantly, offers little information to help prevent these HRI events in the first place. From a causal perspective, two main triggers are described for HRI in humans: exertional and environmental [5]. Exertional HRI typically follows exercise or physical labor in a hot environment or prolonged strenuous exercise in environments of any temperature [5,6]. Environmental HRI, also referred to as classic or non-exertional HRI, typically follows prolonged exposure to high ambient temperatures or shorter exposure to extreme heat [7]. Very young children and babies are similar to dogs in that they are generally not agents of their own liberty from confinement [8]. Very young children and babies are also at risk of vehicular HRI (a subtype of environmental HRI) following confinement in a hot vehicle after being left unattended or after accidentally locking themselves inside the vehicle [8]. In human medicine, exertional HRI most commonly affects young active males either working in physically demanding industries such as construction, or participating in sports [9]. Exertional HRI is the third leading cause of death in US high school athletes, and the incidence of HRI in the US Armed Forces has been gradually increasing since 2014 [10]. Conversely, environmental HRI is known to typically affect socially vulnerable patients, those with advanced age or chronic medical conditions, who may be confined indoors and less resilient to natural hazards such as heatwaves [11,12].

Canine patients are likely to share similar risk factors to humans for the various types of HRI, but there is limited published evidence in the canine literature. Older dogs are more likely to suffer from underlying health conditions that impact thermoregulation such as heart disease [13], which could increase the likelihood of environmental HRI [14]. Respiratory diseases such as brachycephalic obstructive airway disorder (BOAS) have been shown to accelerate the increase in body temperature during exercise [15] and brachycephalic dogs have intrinsically greater odds of developing HRI compared to dogs with longer muzzle [16]. Heat regulation problems are reported to affect around a third of brachycephalic dogs [17] and obesity has been reported as a significant risk factor for death in dogs presenting with HRI [18]. Reflecting the male predisposition to exertional HRI in humans, male dogs develop a significantly higher body temperature than females during intense exercise [19,20]. Both dogs trained for military work (e.g., Belgian Malinois) and active playful dogs (e.g., Golden Retriever and Labrador Retriever) have been reported to be at increased risk of exertional HRI [18], however, that study included only patients referred for specialist care and therefore may not represent the wider canine population. A larger primary-care study reported the Chow Chow and Bulldog with the greatest odds of HRI highlighting the value of primary-care focused research for generalization to the wider companion animal population [21].

Because the veterinary diagnosis of HRI is heavily dependent upon an accurate history of the events leading up to the animal’s presentation [22], greater awareness of the specific risk factors for HRI in dogs could support earlier recognition, diagnosis and appropriate management. However, prevention remains the most important approach to limiting the welfare burden of HRI overall, [23] because irreversible organ damage and cellular destruction follow any occasion when the body is heated beyond 49 °C [5]. Reports from Israel suggest exertional HRI may be the more common cause of heatstroke in dogs in that country [24,25,26]. Although there is some evidence that exertion is also the main trigger of HRI in UK dogs [27], current efforts to educate UK dog owners about heatstroke prevention focus almost exclusively on environmental heatstroke, specifically the message that ‘dogs die in hot cars’ [28]. Generation of a solid evidence base on the predominant trigger of canine HRI, canine risk factors for different HRI types and the seasonality of different HRI types in the UK could support optimized and targeted educational campaigns.

This study aimed to use the VetCompass database of veterinary health records to (i) identify the leading triggers for HRI in the UK general dog population; (ii) identify risk factors for the three key triggers of HRI and (iii) compare the case fatality rate and seasonality between different HRI triggers.

## 2. Materials and Methods

### 2.1. Data Collection and Management

**Figure 1:**
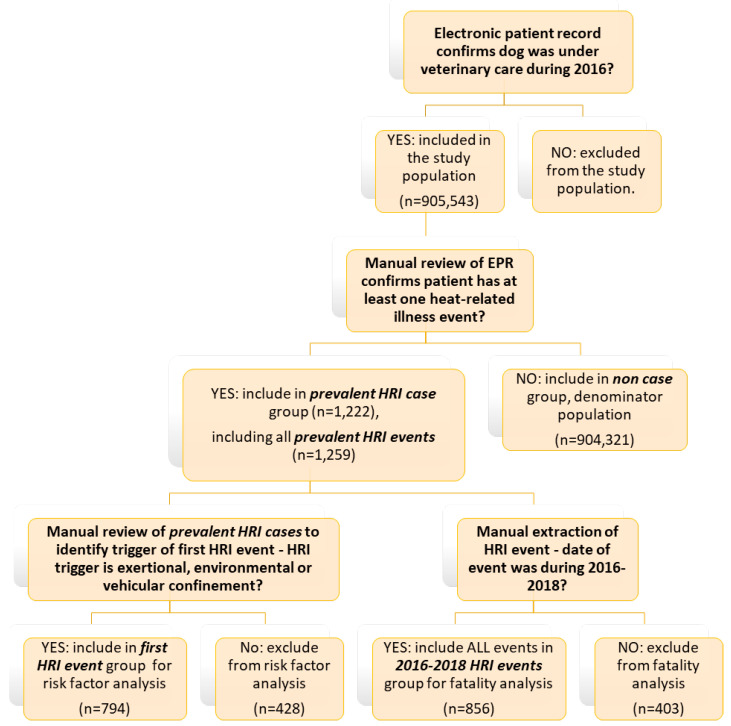
Flow chart of decisions for inclusion in HRI fatality analysis and risk factor analysis for HRI triggers.

This study was an extension of work previously reported in Hall et al. [16] and used the same dataset described in that study. The VetCompass Programme (Royal Veterinary College, London, UK) offers a research database providing access to de-identified electronic patient records (EPRs) from primary-care veterinary practices in the UK [29,30,31,32]. The study population included all dogs under veterinary care during 2016 as previously defined. EPRs were searched to identify candidate cases of HRI, using the following terms: heat stroke~3, heatst*, hyperthermi*, overheat*, over heated~2, heat exhaustion~2, hot car~2, collapse* + heat, cooling, high ambient temp*. Dogs identified from all searches were merged and randomly ordered. All candidate cases were manually reviewed in detail by two researchers (author 1 and author 2) to identify all dogs (confirmed HRI cases) meeting the study case definition for HRI occurring at any date within the patient’s available EPR (prevalent HRI events). All confirmed prevalent HRI events underwent additional data extraction including date of heat exposure event, the trigger for the HRI event (Table 1) and outcome of each event (survival or death). All events were assigned to a single trigger category. For dogs with multiple HRI events, only events occurring between 1st January 2016–31st December 2018 (2016–2018 HRI events) were included when exploring triggers as risk factors for death and reporting numbers of events by trigger. Whilst events occurring after 2016 were excluded from previous risk factor analysis to avoid introducing potential bias from the aging denominator population [16], they were included in the present study to provide greater event numbers for statistical analysis. The earliest HRI event recorded in the EPR (first HRI event) was used to calculate age at the event and was included in risk factor analysis (see Figure 1).

Ethical approval for this study was granted by the RVC Ethics and Welfare Committee (reference number SR2018-1652).

### 2.2. Analysis

The study used a cohort design applied to a denominator population of 905,543 dogs from the VetCompass database under veterinary care in 2016. Demographic data were extracted automatically from the database for all study dogs. Demographic and clinical data were exported into Microsoft Excel (v16, Redmond, WA, USA) for cleaning and descriptive analysis, risk factor analysis used SPSS v25 (IMB Inc., Armonk, NY, USA).

Summary statistics were calculated using all prevalent HRI events to determine the number of HRI events per month and to identify the top three triggers for HRI in UK dogs: exertional HRI, environmental HRI and vehicular HRI. The 2016–2018 HRI events were then assigned to a trigger category for risk factor analysis: exertional HRI, environmental HRI, vehicular HRI, unrecorded HRI, building entrapment, blanket entanglement and undergoing treatment.

#### 2.2.1. Risk Factor Analysis for Fatality

Binary logistic regression modelling was used to compare the odds for death between triggers using only 2016–2018 HRI events, using the most numerous trigger (exertional) as the comparator. Events occurring prior to 2016 were excluded from fatality analysis, because any dogs that died from their HRI event prior to 2016 would by definition be excluded from the denominator population, meaning events prior to 2016 would be biased toward survival.

#### 2.2.2. Risk Factor Analysis for Triggers

Risk factor analysis used cohort clinical data to explore potential canine risk factors for HRI triggered by each of the three key categories: exertional HRI, environmental HRI and vehicular HRI, using all dogs in the denominator population not defined as a confirmed HRI case as the “non-case” population. Where the first HRI event was not triggered by one of the three categories above, these cases were excluded from this analysis as they did not meet the inclusion criteria for “non-case”. As the purpose of this analysis was to identify canine risk factors for the different triggers, events that occurred prior to 2016 were included to increase the number of events available for statistical analysis.

Separate binary logistic regression modelling of the first HRI events was used to identify risk factors for each trigger: exertional HRI, environmental HRI and vehicular HRI. The variables considered in univariable binary logistic regression analysis included breed type, purebred, skull shape, adult bodyweight, bodyweight relative to breed/sex mean, sex/neuter and age. These risk factors were defined in a previous study [16], but are included in Appendix A, Table A1 for reference. Risk factors with liberal associations in univariable modelling (*p* < 0.2) were selected for multivariable evaluation. As breed type was a factor of primary interest, variables that were highly collinear with breed (purebred) or considered a defining characteristic of individual breeds (adult bodyweight and skull shape) were not included in the multivariable models with breed type but were included in alternative models that swapped out the breed type variable as previously described [16,29]. Model development used manual backwards stepwise elimination, as this was an explanatory model aiming to identify canine risk factors, rather than a predictive model [16]. Pairwise interactions were tested for all variables in the final multivariable model. The area under the receiver operating characteristic (ROC) curve was used to evaluate the explanatory ability of the model [38] alongside consideration of the underpinning biological plausibility of the model specification. Statistical significance was set at *p* < 0.05.

## 3. Results

The study included 905,543 dogs under primary veterinary care at 886 UK VetCompass clinics during 2016. From this population, 1259 HRI events were identified from the EPRs of 1222 dogs; 35 dogs had two HRI events and one dog had three HRI events recorded at the time of the study (2.95% of affected dogs had multiple events). Data completeness, incidence estimate and case fatality rate have been reported in a previous study [16].

### 3.1. HRI Triggers

There were 380/1259 (30.2%) prevalent HRI events with no trigger recorded in the EPR. Of the remaining 879 prevalent HRI events, the predominant triggers recorded were exertional HRI (n = 652, 74.2%), followed by environmental HRI (n = 113, 12.9%) and vehicular confinement (n = 46, 5.2%). Other triggers included being under the care of a veterinary clinic or groomer (n = 40, 4.6%), building confinement (n = 24, 2.7%) and blanket entrapment (n = 4, 0.5%).

Of the 652 prevalent HRI events triggered by exercise, the type of exercise was not specified for 101 (15.5%) events. Of the remaining 551 events, 372 (67.5%) occurred after walking, 97 (17.6%) after high-intensity activities such as running or cycling, 76 (13.8%) after periods of play and 6 (1.1%) of those events occurred after canine competitions.

### 3.2. Event Fatality and Seasonality

From the 2016–2018 HRI events, the event fatality rate for each trigger is shown in Table 2. Of the events with a known trigger, building confinement (OR 6.06, 95% CI 2.13–17.22) had greater odds for HRI associated fatality when compared to exertional HRI only. Cases with no recorded trigger also had greater odds (OR 2.43, 95% CI 1.49–3.94).

**Figure 2:**
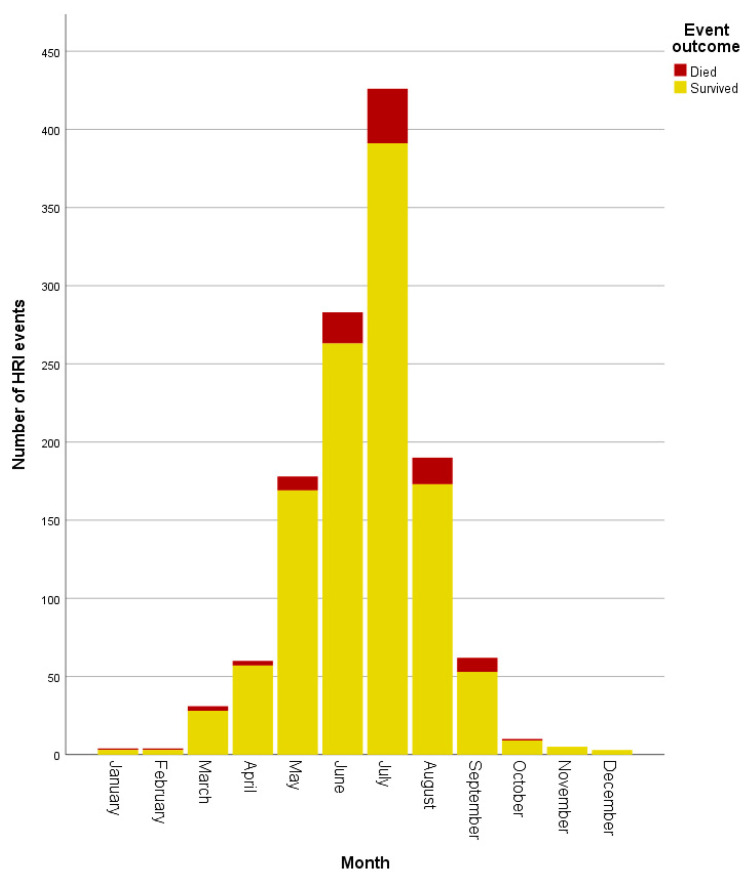
Histogram showing the number of heat-related illness events by outcome per month.

**Figure 3:**
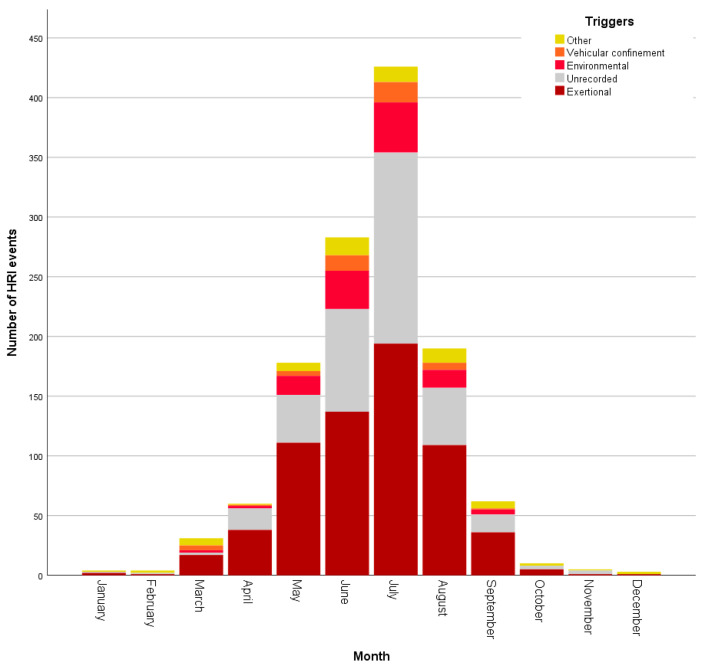
Histogram showing the number of heat-related illness events by a trigger, per month.

HRI events occurred during every month of the year, with July (n = 426, 33.84%) showing the highest proportion of events. Although HRI fatalities were recorded in all months from January to October, July accounted for 35/99 (35.35%) of fatalities (Figure 2). Environmental HRI and vehicular confinement were recorded only between March and September (see Figure 3), corresponding to the UK’s spring to summer period and typically warmest months. Vehicular HRI fatalities only occurred between March and July, whilst environmental HRI fatalities occurred from May to September. Building confinement triggered HRI across all months except November with fatalities occurring from June to September, and undergoing treatment at a vets/groomer triggered HRI in all months except December, January and March with fatalities in June and August. Exertional HRI occurred in all months, however, there was only one exertional HRI fatality (in January) outside the typically warmer UK months of March–October. There were HRI events with no recorded trigger in all months except December, with fatalities from unrecorded triggers occurring between February and September.

### 3.3. Risk Factor Analysis for Exertional HRI

All risk factors were strongly associated with exertional HRI following univariable binary logistic regression modelling (*p* < 0.001). The final breed multivariable model retained four risk factors: breed type, age, bodyweight relative to breed/sex mean and sex/neuter. The model showed acceptable discrimination (R^2^ = 0.004, area under the ROC curve: 0.725). In the final model, seven breeds (Chow Chow, Bulldog, French Bulldog, Greyhound, English Springer Spaniel, Cavalier King Charles Spaniel and Staffordshire Bull Terrier) had higher odds of exertional HRI, and eleven breed types (Crossbred, German Shepherd Dog, West Highland White Terrier, Bichon Frise, Lhasa Apso, Jack Russel Terrier, Designer Crossbred, Shih-tzu, Cocker Spaniel, Yorkshire Terrier and Chihuahua) had lower odds of exertional HRI compared to Labrador Retrievers. As crossbred dogs (previously used as the breed type comparator in other VetCompass studies [30,31,39]) were found to have significantly reduced odds of exertional HRI compared to Labrador Retrievers, the multivariable analysis was repeated using crossbred as the comparator. Labrador Retrievers had 2.1 (95% CI 1.47–2.88) times the odds of exertional HRI compared to non-designer crossbred dogs (the other breeds with significantly greater odds of exertional HRI compared to non-designer crossbred dogs are indicated in Table 3). Dogs aged over 8 years had lower odds of exertional HRI compared to dogs under 2 years of age. Entire females had lower odds of exertional HRI compared to entire males, neutered males and neutered females. Dogs with bodyweight equal to or greater than the breed/sex mean had higher odds of HRI compared to dogs weighing below the relative breed/sex mean (see Table 3).

As described in the methods, variables collinear (purebred) and definitive of breed types (bodyweight and skull shape) replaced the breed type variable in the final multivariable model. Purebred dogs had 1.84 (95% CI 1.47–2.31) times the odds of exertional HRI than crossbred dogs. Dogs in all bodyweight categories over 10 kg had greater odds of exertional HRI compared to dogs under 10 kg bodyweight. Brachycephalic dogs had 1.32 (95% CI 1.10–1.60) times the odds of exertional HRI, and dolichocephalic dogs and brachycephalic crossbred dogs had lower odds of exertional HRI compared to mesocephalic dogs (Appendix B, Table A2).

### 3.4. Risk Factor Analysis for Environmental HRI

Following univariable analysis, all variables were liberally associated with environmental HRI: breed type (*p* = 0.002), age (*p* = 0.051), sex/neuter (*p* = 0.108), purebred (0.054), skull shape (*p* = 0.009), adult bodyweight (*p* = 0.001) and bodyweight relative to breed/sex mean (*p* < 0.001). The final breed multivariable model retained breed type, age and sex/neuter. The final model showed acceptable discrimination (R^2^ = 0.039, area under the ROC curve: 0.721). In the final model, four breeds had higher odds of environmental HRI than Labrador Retrievers: Chow Chow (OR 8.41, 95% CI 1.06–66.70), Bulldog (OR 7.52, 95% CI 2.76–20.47), Pug (OR 3.30, 95% CI 1.16–9.41) and French Bulldog (OR 3.16, 95% CI 1.03–9.73). Dogs aged ≥12 years (OR 3.15, 95% CI 1.56–6.37) had greater odds of environmental HRI compared to dogs <2 years old. Female neutered (OR1.92, 95% CI 1.03–3.56) and male entire (OR1.87, 95% CI 1.05–3.33) had greater odds compared to female entire dogs (Table 4).

Variables collinear (purebred) and definitive of breed types (bodyweight and skull shape) replaced the breed type variable in the final multivariable model. Purebred dogs had 1.97 the odds (95% CI 1.12–3.47) of environmental HRI compared to crossbred dogs. Brachycephalic dogs had 2.36 the odds (95% CI 1.50–3.72) compared to mesocephalic dogs, and dogs in the 10– < 20 kg and 20– < 30 kg bodyweight categories had greater odds for environmental HRI compared to dogs <10 kg (Appendix B, Table A3).

### 3.5. Risk Factor Analysis for Vehicular HRI

Univariable binary logistic regression modelling identified breed type (*p* = 0.007), sex/neuter (*p* = 0.199) and skull shape (*p* = 0.007), as factors liberally associated with vehicular HRI, but not age (*p* = 0.690), bodyweight relative to breed/sex mean (*p* = 0.269), purebred (*p* = 0.395) or bodyweight (*p* = 0.245). The final breed multivariable model retained two risk factors: breed type and sex/neuter. The model showed good discrimination (R^2^ = 0.066, area under the ROC curve: 0.808). In the final model (Table 5) five breeds had increased odds of vehicular HRI compared to the Labrador Retriever: Bulldog (OR 16.63, 95% CI 3.02–91.53), Greyhound (OR 9.59, 95% CI 1.35–68.23), Cavalier King Charles Spaniel (OR 8.52, 95% CI 1.65–43.94), French Bulldog (OR 6.70, 95% CI 1.11–40.65) and Pug (OR 6.29, 95% CI 1.05–37.84) (Table 5).

Variables definitive of breed types (skull shape) replaced the breed type variable in the final multivariable model. Brachycephalic dogs had greater odds (OR 3.07, 95% CI 1.60–5.87) of vehicular HRI compared to mesocephalic dogs (see Appendix B, Table A4).

## 4. Discussion

This is the largest primary-care study worldwide to deconstruct and explain HRI triggers in companion dogs. Reflecting the results of previous studies from Israel [24,25,26], the predominant trigger of HRI presenting to UK primary-care practices was exertional HRI (74.2% of events with a known cause). Exertional HRI events occurred year-round, with a 7.6% overall fatality rate. Exertional HRI accounted for the majority of HRI related deaths with known triggers. The odds of death did not differ between exertional HRI, environmental HRI or vehicular HRI.

Seven breed types had greater odds of exertional HRI when compared to Labrador retrievers: Chow Chow, Bulldog, French Bulldog, Greyhound, English Springer Spaniel, Cavalier King Charles Spaniel and Staffordshire Bull Terrier. Six of those breeds have previously been identified with greater odds for HRI in general [16], whereas the Staffordshire Bull Terrier appears to have greater odds specifically for exertional HRI. The Labrador Retriever was chosen as the comparator breed for reasons highlighted previously [16,40] namely that the Labrador Retriever was the most common definitive breed type in the study population. However, the Labrador Retriever had twice the odds for exertional HRI compared to non-designer crossbred dogs, as did several other large active breeds (Boxer, Golden Retriever and Border Collie), along with the Pug, and “other purebred”—namely breeds with either relatively low numbers in the study population or fewer than five confirmed HRI cases. Labrador Retrievers, Golden Retrievers, Border Collies and English Springer Spaniels represent breeds that are commonly used as working or assistance/service dogs including guide dogs for the visually impaired, hearing assistance dogs, medical support dogs, military detection dogs and medical detection dogs. Given the current evidence that these breeds show increased risk of exertional HRI, it is essential that future societal adaptations to increasing ambient temperature include appropriate mitigations to safeguard working and assistance dogs.

German Shepherd Dogs showed just one-third of the odds for exertional HRI compared to the Labrador Retriever in the current study. Both Australian Shepherd Dogs [34] and Belgian Malinois [18] have previously been identified with increased risk of exertional HRI, however, these studies used referral hospital populations and thus likely included a relatively higher proportion of military or police working dogs than the present study based on the general population of dogs. German Shepherd Dogs and Belgian Malinois comprised over 70% of the US civilian law enforcement dogs in one study [41], in which HRI was the second most common cause of traumatic death accounting for approximately a quarter of deaths. However, 75% of those HRI events were triggered by vehicular confinement. The conflicting findings of this study compared to previous reports suggesting an increased risk of exertional HRI in Shepherd type dogs likely reflects the difference in study populations, with the current study population being the first to explore HRI in first opinion veterinary practice. Additionally, the potential for an underlying genetic predisposition for HRI in military working dogs (Belgian Malinois) has been suggested [42], potentially associated with low levels of expression of heat shock proteins [43].

Exertional HRI appears to predominantly affect younger dogs, all age groups of dogs over 8 years had reduced odds compared to dogs less than 2 years of age. This may reflect differing intensity and duration of exercise undertaken by younger dogs whereas older dogs are more likely to suffer from conditions that limit their ability to exercise, such as osteoarthritis [30] and cardiac disease [44]. Male dogs and neutered female dogs had greater odds than entire female dogs for exertional HRI. These findings mirror the human risk factors of exertional HRI, with young male athletes and labourers most likely to be affected [45]. Entire female dogs could have reduced odds for exertional HRI due to their relatively lower bodyweights compared to male and neutered animals [19], or, it could reflect reduced exercise levels during reproductive periods such as pregnancy and lactation. Dogs at or above the mean adult bodyweight for their breed/sex showed an increased risk of exertional HRI compared to dogs below the mean bodyweight, and all dogs weighing 10 kg or over had increased odds of exertional HRI compared to dogs weighing under 10 kg.

Although the precise mechanisms behind the differing odds between categories for exertional HRI was not explored in this study, it is important to note that HRI is a disorder that requires extrinsic (and often human-controlled) input—dogs cannot develop HRI without exposure to a hot environment or exercise that results in overwhelming hyperthermia [16]. Exertional HRI requires the dog to have undertaken either exercise in a hot environment [6], or prolonged or intense exercise sufficient to exceed thermoregulatory capacity. The majority of exertional HRI events in the present study occurred following relatively low-intensity activities such as walking and occurred during the typically warmer spring and summer months. However, as demonstrated in Figure 3, exertional HRI events occurred in every month (albeit with lower numbers between October and February), confirming that exertional HRI is a year-round risk for UK dogs.

Several breeds along with both non-designer crossbred and designer crossbred dogs were identified with reduced odds for HRI compared to the Labrador Retriever. Conversely, only Chihuahuas were identified with reduced odds (OR 0.42, 95% CI 0.20–0.92) of exertional HRI compared to non-designer crossbred dogs. The Chihuahua is the smallest breed of dog in the world [46], with owners reportedly more influenced by “convenience” when choosing this breed than owners of other popular dog breeds [47]. Chihuahua ownership and popularity are also reported to be influenced by fashion and celebrity trends, with the breed frequently depicted as a “handbag dog” being carried as a fashion statement [48]. Their relatively smaller bodyweight is likely to confer a degree of protection from HRI as previously identified [16,49], which could potentially be augmented by their greater likelihood of being carried than other breeds which could reduce the risk of exertional HRI due to reduced exercise.

Dogs with both dolichocephalic and brachycephalic-cross skull shapes showed reduced odds of exertional HRI, whilst brachycephalic dogs had increased odds of exertional HRI when compared to mesocephalic dogs. This gradient of increasing exertional HRI risk with shortening of the skull is likely due to the differing relative surface area of the nasal turbinates. Evaporative heat loss from panting and respiration is an important aspect of canine thermoregulation [50], so, therefore, dogs with longer muzzles have more surface area for evaporative heat loss. The reduced odds of exertional HRI for brachycephalic-crosses is unexpected but may reflect the diversity of skull conformation types within this ill-defined category. This group is likely to be younger compared to the rest of the study population [16], however, younger dogs have increased odds of exertional HRI. The group is also likely to have relatively lower bodyweight compared to both purebred and non-designer crossbred dogs [16], but could also be subject to similar lifestyle differences, e.g., “handbag dogs”, as the Chihuahua due to their small stature and “designer” status.

The second most commonly reported HRI trigger was environmental (12.9% of events with a known cause). Environmental triggers were only recorded between March and September, reflecting the UK’s warmer season. The four breeds with increased odds of environmental HRI when compared to the Labrador Retriever were predominantly brachycephalic breeds (Bulldog, Pug and French Bulldog); brachycephalic dogs, in general, had 2.36 the odds compared to mesocephalic breeds. This mirrors the increased risk of environmental HRI for humans with underlying respiratory disorders, and is supported by the findings of Lilja-Maula et al. [15] that documented Bulldogs developing hyperthermia just standing in ambient room temperature (21 °C). Although the Chow Chow had the greatest odds of both exertional and environmental HRI, it must be noted that the Chow Chow breed group was the smallest in the study population resulting in very wide confidence intervals, and so these results need to be generalized to the wider population with caution.

Dogs aged 12 years or over had over three times the odds of environmental HRI compared to dogs under 2 years, again mirroring human risk factors. Advancing age increases the likelihood of underlying health conditions such as cardiac or respiratory disease, and old age in humans has been shown to increase HRI risk due to decreased physiological thermoregulatory mechanisms such as decreased sweat production and skin blood flow [14,51]. Dogs weighing from 10 up to 30 kg had almost twice the odds of environmental HRI compared to dogs weighing less than 10 kg, however, interestingly none of the dogs weighing 50 kg or over were reported with environmental HRI. In general, the risk factor analysis for environmental HRI was the least informative of the three models, with the lowest R^2^ and area under the ROC curve values. Environmental HRI requires prolonged exposure to a hot environment, or acute exposure to an extremely hot environment, both traditionally rare events in the UK. Environmental HRI is also the trigger for dogs that is least influenced by human behaviour, whereas both exertional HRI and vehicular HRI are heavily dependent on the actions of the dog’s owner. The much lower levels of environmental HRI compared with exertional HRI offers a substantial welfare gain opportunity by empowering owners with management tools that can limit the exertional HRI risk to their dogs. However, if climate change continues to increase, the frequency of heatwave events in the UK, the number of dogs experiencing environmental HRI is likely to increase without appropriate mitigation strategies.

Five breed types had increased odds of vehicular HRI compared to the Labrador Retriever (Bulldog, Greyhound, Cavalier King Charles Spaniel, French Bulldog and Pug). Brachycephalic dogs overall had three times the odds compared to mesocephalic dogs. However, the relatively low number of vehicular HRI events (37/856) resulted in low statistical power for this analysis, as reflected in the wide confidence intervals. Only two variables remained in the final vehicular HRI risk factor model, likely reflecting the predominantly extrinsic causal structure to vehicular HRI. Any dog subjected to confinement in a hot car will overheat, as their thermoregulatory mechanisms cease to be effective once ambient temperature exceeds body temperature. Internal car temperature in the UK can exceed 50 °C between May and August, and can exceed 40 °C between April and September [52]. The duration of confinement and the temperature within the vehicle will determine the severity of HRI [53], however underlying canine factors that impact thermoregulatory ability (such as a respiratory compromise or disease [50], acclimatization [54,55,56] and hydration [57]) will result in dogs overheating and developing HRI at lower relative temperatures.

Vehicular HRI was the third most common trigger and was reported only between March and September. Welfare charities and UK veterinary organisations run an annual “Dogs die in hot cars campaign”, traditionally launched around May [58,59]. However, Carter et al. [52], report that internal vehicle temperatures exceeded 35 °C between the months of April and September in a study measuring UK vehicle temperatures for a two-year period. As heat acclimatization is known to impact susceptibility to HRI, sudden warm spells in March and April may be particularly dangerous for dogs left in cars. The findings of the current study, and those of Carter et al. [52], support an earlier launch of this annual awareness campaign.

There was no significant difference in the odds for HRI related fatality between vehicular and exertional HRI. However, because exertional HRI affected around ten times as many dogs and resulted in eight times as many deaths overall than vehicular HRI, there is now a strong evidential basis to suggest that educational campaigns aimed at owners need to move from focusing purely on the risk of vehicular HRI to dogs and instead to include warnings about the more frequent dangers of exercising in hot weather.

This study identifies some important novel HRI triggers, in particular dogs developing HRI whilst under the care of veterinary practices and professional groomers. Undergoing treatment at a veterinary practice or grooming parlour was the fourth most common trigger for HRI events with a known cause, with a similar fatality rate to both exertional and vehicular HRI. This topic was explored further as part of an abstract presentation, reporting that two-thirds of the HRI events occurred in a veterinary practice (56% brachycephalic dogs) and one third whilst dogs were undergoing professional grooming (45% brachycephalic) [27]. The French Bulldog and the Bulldog accounted for a third of the cases occurring under veterinary care, whilst West Highland White Terriers were the most numerous breed type affected during grooming. Both veterinary practice premises and grooming parlours can be warm, stressful environments for dogs, highlighting the need for careful patient monitoring and awareness of the risk of HRI in these situations, especially in predisposed breeds.

Other HRI triggers identified in the current study included building confinement and blanket entrapment. These two triggers had the highest fatalities rates, with building confinement resulting in HRI all year round. Building confinement (OR 6.1) and unrecorded trigger (OR 2.4) HRI events both had significantly greater odds for HRI fatality compared to exertional HRI. Building confinement HRI events included events where central heating had been accidentally left on, or developed a fault, and so resulted in dogs being restricted to hot environments for prolonged periods while owners were unaware of the problem. The HRI events with unrecorded triggers included emergency presentations where the attending veterinary surgeon potentially did not have time to accurately record a history in the EPR and also includes HRI events where a specific trigger was not recognised or reported by the owner. Increasing owner awareness of circumstances that can result in canine HRI should be a priority as global temperatures continue to rise.

Dogs have been proposed as an ideal translational model for studying human morbidity and mortality [4,60]. Domestic dogs often share their owners’ home and leisure activities including walking, running and other sports [20]. Dogs increasingly accompany their owners to the workplace [61] and are often included in travel and holiday plans. No other species more intimately intertwines with the human lifestyle, meaning dogs potentially face similar levels of both environmental and exertional heat exposure to humans. The results of the current study highlight that dogs share similar risk factors to humans for both exertional and environmental HRI. How dogs are transported, housed and managed will also influence HRI risk. Dogs housed outside, with no access to air conditioning or fans will be at increased risk of environmental HRI as global warming worsens. The vehicular HRI events in the current study included both dogs left in parked vehicles and dogs travelling in hot vehicles, and highlight the danger of transporting dogs in cars without adequate ventilation or air conditioning during hot weather. As the frequency of extreme weather events such as heat waves is increasing, society needs to prepare strategies to mitigate the threat of HRI [62], to protect both human and canine health [22].

This study had some limitations. As previously reported, the clinical record data in the VetCompass programme were not recorded primarily for research purposes, meaning there are missing data within the dataset and the accuracy of descriptive entries (such as patient histories recording HRI triggers) is reliant upon the history provided to the veterinary surgeon treating the animal and their clinical note-taking [31,63]. Other limitations including the lack of a definitive diagnostic test for HRI, the use of skull shape definitions such as brachycephalic and mesocephalic, and the use of manual stepwise elimination to select the final breed models for the various HRI triggers have been discussed in a previous study [16].

The present study used prevalent HRI events recorded at any point within the available clinical records for each dog. This may have selectively biased towards less severe HRI events, because dogs that died as a result of HRI prior to 2016 were by definition not part of the study population. This is reflected in the overall fatality rate of the prevalent cases in the present study (7.86%) which is lower than the 2016 incident fatality rate (14.18%) reported previously [16]. As the main aims of the present study were to identify the predominant triggers for HRI in UK dogs, and explore risk factors for the top three triggers, the decision to include all prevalent HRI events was made to increase the number of events available for analysis, and thus improve the statistical power of the findings.

Finally, the present study aimed to identify potential risk factors for different HRI triggers, producing potentially explanatory models, rather than predictive models. The low R^2^ values for all three risk factor models highlight the impact of non-canine variables as important driving forces for HRI in dogs. The effect of ambient temperature and humidity, canine behaviour and activity status, heat acclimation, athletic fitness and overall health fitness would all need to be considered to create a truly predictive model for canine HRI. These variables are not recorded in veterinary EPRs, meaning it was not possible to include these factors in the present analysis.

## 5. Conclusions

This study highlights canine risk factors for the three most common triggers of HRI in UK dogs, providing both dog owners and veterinary professionals information that can be used to identify at-risk dogs, tailor HRI education and potentially assist with more rapid recognition and therefore treatment of HRI in dogs. Dogs appear to share similar risk factors to humans for both of the most common HRI triggers: exertional and environmental. Young, active male dogs appear to have the greatest odds for exertional HRI, older dogs and brachycephalic dogs have greater odds for environmental HRI. Exertional HRI is shown to result in almost ten times the health welfare burden for dogs compared with vehicular HRI. It is hoped that these results will help to inform more targeted education campaigns, and catalyse further research to develop canine HRI mitigation strategies in the face of increasing global temperatures.

## Figures and Tables

**Table 1:** Definitions of heat-related illness (HRI) triggers in UK dogs.

Trigger	Definition	Justification
Exertional	Any HRI event where physical activity is identified as the inciting cause regardless of ambient temperature. All types of physical activity are included.	Exertional HRI is reported to affect both humans [10], horses [33] and dogs [22].
Environmental	Any HRI event where the dog was reported to be outdoors in the heat but not exercising.	Environmental HRI typically affects dogs in the summer months, but can also be triggered by unseasonable hot spells especially when the dog has not been acclimatized to heat [18,34].
Undergoing treatment (vets/groomer)	HRI events that occurred following hospitalisation in a veterinary clinic, or following professional grooming.	Frenzied jumping and barking has been reported to trigger HRI in dogs [34], and can occur following separation from owners in veterinary clinics. Dogs have been reported to suffer HRI following grooming in media reports [35].
Blanket entrapment	HRI events resulting from the dog becoming entangled in blankets.	Wearing thick or insulating clothing increases the risk of HRI in humans [12].
Building confinement	HRI events that occurred following confinement in a hot building.	Confinement indoors with no air conditioning is a known risk factor for elderly human HRI patients [12].
Vehicular confinement	HRI events that occurred following confinement in a hot vehicle, including both dogs left unattended in a hot vehicle and dogs travelling in a hot vehicle.	Enclosed vehicles can reach 54.4 °C [36] in the summer months. Vehicular confinement was the leading trigger of HRI in dogs presenting to a German veterinary hospital [37].
Unrecorded	Confirmed HRI events with no record of an inciting cause in the clinical history.	HRI requires urgent treatment, meaning some dogs present as emergencies needing immediate triage and treatment. Clinical notes may not be written at the time of presentation limiting the information recorded. Additionally, some owners may be reluctant to disclose the inciting cause or may not have recognised the condition as HRI.

**Table 2:** Descriptive and binary logistic regression results for triggers as risk factors for heat-related illness associated fatality in dogs affected between 2016–2018, under primary veterinary care in the VetCompass programme in the UK during 2016.

Trigger	Number of Events (n = 856)	% of Known Events (n = 592)	Event Fatality (Rate)	Odds Ratio	95% Confidence Interval	*p*-Value	
Exertional	420	70.9%	32 (7.6%)	base			
Blanket entrapment	2	0.3%	1 (50.0%)	12.13	0.74–198.43	0.080	
Building confinement	18	3.0%	6 (33.3%)	6.06	2.13–17.22	0.001	
Environmental	84	14.2%	8 (9.5%)	1.28	0.57–2.88	0.556	
Undergoing treatment (vet/groomer)	31	5.2%	3 (9.7%)	1.3	0.37–4.51	0.680	
Unrecorded	264		44 (16.7%)	2.43	1.49–3.94	<0.001	
Vehicular confinement	37	6.3%	4 (10.8%)	1.47	0.49–4.41	0.492	

**Table 3:** Multivariable binary logistic regression results for risk factors associated with exertional heat-related illness in dogs under primary veterinary care in the VetCompass programme.

Risk Factor	Category	Odds Ratio	95% CI	*p*-Value
Breed type	Labrador Retriever *	Base		
	Chow Chow *	7.00	3.00–16.33	<0.001
	Bulldog *	3.73	2.34–5.93	<0.001
	French Bulldog *	2.96	1.96–4.47	<0.001
	Greyhound *	2.11	1.08–4.16	0.03
	English Springer Spaniel *	1.85	1.21–2.84	0.005
	Cavalier King Charles Spaniel *	1.69	1.06–2.67	0.026
	Staffordshire Bull Terrier *	1.56	1.09–2.22	0.015
	Pug *	1.58	0.97–2.56	0.064
	Boxer *	1.31	0.68–2.50	0.418
	Golden Retriever *	1.25	0.65–2.39	0.499
	Border Collie *	1.15	0.71–1.88	0.566
	Dogue de Bordeaux	1.12	0.35–3.59	0.848
	Missing	1.09	0.26–4.57	0.909
	Pomeranian	1.08	0.46–2.51	0.86
	Rottweiler	0.96	0.42–2.24	0.931
	Border Terrier	0.89	0.43–1.88	0.764
	Other purebred *	0.73	0.52–1.03	0.077
	Siberian Husky	0.61	0.19–1.95	0.405
	Cockapoo	0.52	0.26–1.02	0.056
	Miniature Schnauzer	0.49	0.18–1.35	0.168
	Lurcher	0.37	0.09–1.52	0.167
	Beagle	0.25	0.06–1.01	0.052
	Yorkshire Terrier	0.52	0.29–0.96	0.036
	Cocker Spaniel	0.52	0.30–0.91	0.022
	Non-designer Crossbred	0.49	0.35–0.68	<0.001
	Jack Russell Terrier	0.43	0.25–0.73	0.002
	West Highland White Terrier	0.43	0.20–0.94	0.034
	German Shepherd Dog	0.33	0.14–0.76	0.01
	Labradoodle	0.26	0.06–1.06	0.06
	Shih-tzu	0.25	0.12–0.53	<0.001
	Chihuahua **	0.21	0.09–0.45	<0.001
	Other designer crossbred	0.19	0.07–0.52	0.001
	Lhasa Apso	0.17	0.04–0.69	0.013
	Bichon Frise	0.16	0.04–0.64	0.01
Age	<2 years	Base		
	2– < 4 years	1.23	0.97–1.56	0.09
	4– < 6 years	0.82	0.62–1.08	0.157
	6– < 8 years	0.77	0.57–1.03	0.08
	8– < 10 years	0.48	0.33–0.69	<0.001
	10– < 12 years	0.48	0.32–0.72	<0.001
	≥12 years	0.54	0.36–0.81	0.002
	Unrecorded	1.37	0.63–2.98	0.424
Sex/neuter	Female-entire	Base		
	Female-neutered	1.63	1.26–2.10	<0.001
	Male-entire	1.49	1.18–1.88	<0.001
	Male-neutered	1.83	1.43–2.33	<0.001
	Unrecorded	0	~	0.982
Bodyweight relative to breed/sex mean	Lower	Base		
	Equal/Higher	1.30	1.09–1.55	0.003
	Unrecorded	0.49	0.30–0.62	<0.001

* Indicates breeds with significantly greater odds of exertional HRI compared to non-designer crossbred dogs. ** Indicates breeds with significantly reduced odds of exertional HRI compared to non-designer crossbred dogs.

**Table 4:** Multivariable binary logistic regression results for risk factors associated with environmental heat-related illness in dogs under primary veterinary care in the VetCompass programme.

Risk Factor	Category	Odds Ratio	95% CI	*p*-Value
Breed type	Labrador Retriever	Base		0.002
	Chow Chow	8.41	1.06–66.70	0.044
	Bulldog	7.52	2.76–20.47	<0.001
	Pug	3.30	1.16–9.41	0.025
	French Bulldog	3.16	1.03–9.73	0.045
	Cavalier King Charles Spaniel	2.38	0.85–6.69	0.100
	Boxer	2.22	0.60–8.19	0.233
	Other designer crossbred	1.33	0.36–5.01	0.67
	Jack Russell Terrier	1.29	0.52–3.17	0.583
	West Highland White Terrier	1.21	0.37–3.96	0.748
	Bichon Frise	1.08	0.23–5.00	0.923
	Labradoodle	1.06	0.13–8.38	0.958
	Beagle	0.95	0.12–7.48	0.958
	Staffordshire Bull Terrier	0.88	0.33–2.36	0.798
	Cocker Spaniel	0.88	0.27–2.85	0.829
	Chihuahua	0.71	0.19–2.65	0.612
	Yorkshire Terrier	0.70	0.19–2.59	0.594
	Other purebred	0.69	0.29–1.64	0.404
	German Shepherd Dog	0.69	0.15–3.18	0.629
	Border Terrier	0.67	0.09–5.28	0.703
	Golden Retriever	0.65	0.08–5.11	0.680
	Border Collie	0.58	0.13–2.70	0.490
	Lhasa Apso	0.55	0.07–4.34	0.571
	Cockapoo	0.50	0.06–4.01	0.515
	Non-designer Crossbred	0.50	0.22–1.16	0.105
	Shih-tzu	0.46	0.10–2.13	0.320
	English Springer Spaniel	0.00	~	0.965
	Miniature Schnauzer	0.00	~	0.977
	Rottweiler	0.00	~	0.979
	Pomeranian	0.00	~	0.981
	Lurcher	0.00	~	0.981
	Greyhound	0.00	~	0.981
	Siberian Husky	0.00	~	0.982
	Dogue de Bordeaux	0.00	~	0.987
	Missing	0.00	~	0.984
Age	<2 years	Base		0.081
	2– < 4 years	1.35	0.72–2.50	0.349
	4– < 6 years	1.09	0.53–2.24	0.821
	6– < 8 years	1.67	0.84–3.35	0.146
	8– < 10 years	1.81	0.88–3.73	0.110
	10– < 12 years	1.64	0.71–3.76	0.244
	≥12 years	3.15	1.56–6.37	0.001
	Unrecorded	0.00	~	0.973
Sex/neuter	Female-entire	Base		0.373
	Female-neutered	1.92	1.03–3.56	0.039
	Male-entire	1.87	1.05–3.33	0.033
	Male-neutered	1.72	0.93–3.21	0.086
	Unrecorded	0.00	~	0.984

**Table 5:** Multivariable binary logistic regression results for risk factors associated with vehicular heat-related illness in dogs under primary veterinary care in the VetCompass Programme.

Risk Factor	Category	Odds Ratio	95% CI	*p*-Value
Breed type	Labrador Retriever	Base		
	Bulldog	16.63	3.02–91.53	0.001
	Greyhound	9.59	1.35–68.23	0.024
	Cavalier King Charles Spaniel	8.52	1.65–43.94	0.010
	French Bulldog	6.70	1.11–40.65	0.039
	Pug	6.29	1.05–37.84	0.045
	Siberian Husky	6.10	0.55–67.40	0.140
	Pomeranian	5.31	0.48–58.76	0.173
	Labradoodle	3.88	0.35–42.83	0.268
	Boxer	3.22	0.29–35.49	0.340
	Border Terrier	3.07	0.28–33.84	0.360
	Golden Retriever	3.05	0.28–33.63	0.363
	Cocker Spaniel	2.82	0.47–16.90	0.256
	Shih-tzu	1.89	0.27–13.46	0.523
	Cockapoo	1.70	0.15–18.79	0.664
	West Highland White Terrier	1.51	0.14–16.62	0.738
	German Shepherd Dog	1.49	0.14–16.48	0.744
	Border Collie	1.33	0.12–14.65	0.817
	Jack Russell Terrier	1.21	0.17–8.58	0.850
	Non-designer Crossbred	0.90	0.18–4.47	0.901
	Chihuahua	0.89	0.08–9.83	0.923
	Staffordshire Bull Terrier	0.58	0.05–6.39	0.655
	Other purebred	0.50	0.07–3.55	0.489
	Bichon Frise	0.00	~	0.975
	Lhasa Apso	0.00	~	0.976
	English Springer Spaniel	0.00	~	0.969
	Miniature Schnauzer	0.00	~	0.980
	Beagle	0.00	~	0.980
	Rottweiler	0.00	~	0.981
	Lurcher	0.00	~	0.983
	Dogue de Bordeaux	0.00	~	0.988
	Other designer crossbred	0.00	~	0.969
	Missing	0.00	~	0.986
	Yorkshire Terrier	0.00	~	0.963
	Chow Chow	0.00	~	0.993
Sex/neuter	Female-entire	Base		
	Female-neutered	1.49	0.69–3.22	0.316
	Male-entire	0.43	0.16–1.13	0.086
	Male-neutered	1.27	0.58–2.79	0.546
	Unrecorded	0.00	~	0.984

## Data Availability

https://researchonline.rvc.ac.uk/id/eprint/12745/.

## Appendix A

**Table A1:** Potential canine risk factors for association with HRI in UK dogs [16].

Potential Risk Factor for HRI	Variable Definition	Justification
Breed type	Categorical variable including all named breed types (including both KC recognised purebred and non-KC recognised purebred) and designer hybrid types with contrived names (e.g., Cockapoo, Labradoodle, Lurcher) with ≥5 HRI cases and/or ≥5000 dogs in the overall study population. All remaining dogs were assigned to grouped categories of “other purebred”, “other designer cross” or “non-designer crossbred”.	Chow Chow, Bulldog, French Bulldog, Dogue de Bordeaux, Greyhound, Cavalier King Charles Spaniel, Pug, English Springer Spaniel and Golden Retriever breeds have all be identified as having greater odds of HRI in UK dogs [16]. Labrador Retriever was used as the comparator for this variable as they were the largest breed type in the denominator population (after crossbred) so enabled high statistical power to explore breed risks [38,40].
Purebred	Categorical variable grouping all dogs of recognisable breeds as “purebred”, all recognisable designer crossbreeds as “designer cross” and the remaining dogs as “crossbred”.	Purebred dogs are more likely to have an exaggerated conformation such as brachycephaly, thick coat, or giant body size, limiting their ability to thermoregulate [50]. A higher percentage of purebred dogs presented with heatstroke to one veterinary hospital [34].
Skull shape	Purebred dogs were categorised by skull shape into three groups, “brachycephalic”, “mesocephalic” and dolichocephalic” (see Supplementary note 1 for breeds by category). Designer crossbred dogs including a brachycephalic breed were classified as “brachycephalic cross” and all other dogs listed as crossbred or unrecorded breed were classified as “skull shape unrecorded”.	Surface areas of the nasal turbinates and effective ventilation provide the mechanism to enable evaporative heat loss through panting, thus brachycephalic dogs have reduced heat dissipation mechanisms [15,18,50,64].
Adult bodyweight	Adult bodyweight was defined as the mean of all bodyweight (kg) values recorded for each dog after reaching 18 months old. Bodyweight (kg) was then categorised into seven groups (<10, 10– < 20, 20– < 30, 30– < 40, 40– < 50, ≥50), dogs under 18 months or with no recorded adult bodyweight were classified as “unrecorded”.	Small breeds of dog are reported to have decreased risk of HRI [18], dogs with greater body mass have been reported to develop higher post exercise body temperatures [19].
Bodyweight relative to breed/sex mean	A categorical variable grouping dogs with a mean adult bodyweight “equal or above” or “below” the mean adult bodyweight for their breed and sex (calculated using the overall VetCompass study population). An “unrecorded” variable included all dogs with no adult bodyweight or labelled as crossbred.	Increased bodyweight can be due to increases in either lean muscle mass, or body fat. Obesity limits heat conduction and radiation from the skin and can limit effective cooling via respiration [50], overweight animals overheat faster and take longer to cool [65]. Dogs with greater lean body mass developed higher post-exercise temperatures than lighter dogs [19].
Sex/neuter	Dogs were classified by sex and neuter status into five categories (female entire, female neutered, male entire, male neutered) with “unrecorded” was used to group any dogs with no recorded sex or neuter status.	Male dogs develop higher body temperature post exercise [19,20], and are over-represented in cases of heatstroke presenting to veterinary hospitals [37,66,67].
Age	The age variable described the age of the dog at the end of the study period (31st December 2016) for non-case dogs, or the age at the first HRI event for 2016 incident HRI cases. Age (years) was categorised into eight groups (<2, 2– < 4, 4– < 6, 6– < 8, 8– < 10, 10– < 12, ≥12) with “unrecorded” for any dogs with no date of birth recorded in the EPR.	Older animals are more likely to have pre-existing conditions that limit effective heat dissipation such as heart disease, or respiratory diseases e.g., laryngeal paralysis [64].

## Appendix B

**Table A2:** Results for variables that individually replaced the breed type variable in the final multivariable logistic regression model (with age, bodyweight relative to breed/sex mean and sex/neuter) to evaluate risk factors associated with exertional heat-related illness in dogs under primary veterinary care in the VetCompass Programme.

Risk Factor	Category	Odds Ratio	95% CI	*p*-Value
Purebred	Non-designer Crossbred	Base		
	Designer Crossbred	0.68	0.41–1.13	0.134
	Purebred	1.84	1.47–2.31	<0.001
	Unrecorded	2.11	0.51–8.78	0.306
Skull shape	Mesocephalic	Base		
	Dolichocephalic	0.72	0.52–1.00	0.048
	Brachycephalic-cross	0.19	0.05–0.75	0.018
	Brachycephalic	1.32	1.10–1.60	0.004
	Unknown	0.60	0.47–0.76	<0.001
Adult bodyweight (kg)	<10 kg	Base		
	10– < 20 kg	2.52	1.97–3.22	<0.001
	20– < 30 kg	2.27	1.73–2.99	<0.001
	30– < 40 kg	2.30	1.68–3.15	<0.001
	40– < 50 kg	2.15	1.31–3.53	0.002
	≥50 kg	2.21	1.02–4.79	0.046
	Unrecorded	0.54	0.13–2.23	0.395

**Table A3:** Results for variables that individually replaced the breed type variable in the final multivariable logistic regression model (with age and sex/neuter) to evaluate risk factors associated with environmental heat related illness in dogs under primary veterinary care in the VetCompass Programme.

Risk Factor	Category	Odds Ratio	95% CI	*p*-Value
Purebred	Non-designer Crossbred	Base		
	Designer Crossbred	1.55	0.55–4.34	0.404
	Purebred	1.97	1.12–3.47	0.018
	Unrecorded	0.00	~	0.985
Skull shape	Mesocephalic	Base		
	Dolichocephalic	0.81	0.37–1.79	0.606
	Brachycephalic-cross	1.94	0.46–8.16	0.364
	Brachycephalic	2.36	1.50–3.72	<0.001
	Unknown	0.61	0.34–1.09	0.094
Adult bodyweight (kg)	<10 kg	Base		
	10– < 20 kg	1.82	1.07–3.08	0.027
	20– < 30 kg	1.90	1.04–3.45	0.036
	30– < 40 kg	1.61	0.78–3.32	0.200
	40– < 50 kg	1.52	0.45–5.09	0.498
	≥50 kg	0.00	~	0.980
	Unrecorded	0.56	0.29–1.06	0.074

**Table A4:** Results for variables that individually replaced the breed type variable in the final multivariable logistic regression model (with sex/neuter) to evaluate risk factors associated with vehicular heat related illness in dogs under primary veterinary care in the VetCompass Programme.

Risk Factor	Category	Odds Ratio	95% CI	*p*-Value
Skull shape	Mesocephalic	Base		
	Dolichocephalic	1.00	0.29–3.39	0.999
	Brachycephalic-cross	0.00	~	0.975
	Brachycephalic	3.07	1.60–5.87	0.001
	Unknown	0.74	0.29–1.86	0.522
